# Does body weight support improve neural and biomechanical measures during treadmill gait in children with unilateral cerebral palsy?

**DOI:** 10.3389/fresc.2025.1607515

**Published:** 2025-10-23

**Authors:** Rajit Banerjee, Yushin Kim, Thomas C. Bulea, Diane L. Damiano

**Affiliations:** ^1^ Rehabilitation Medicine Department, Neurorehabilitation and Biomechanics Research Section, National Institutes of Health Clinical Center, Bethesda, MD, United States; ^2^ Department of Physical Medicine and Rehabilitation, University of Pittsburgh Medical Center, Pittsburgh, PA, United States; ^3^ Department of Sports Rehabilitation, Cheongju University, Cheongju, Republic of Korea

**Keywords:** muscle synergies, electroencephalography, kinematics, temporal-spatial, non-negative matrix factorization, unloading

## Abstract

**Introduction:**

Body weight support (BWS) treadmill training, commonly utilized to improve gait, has inconsistent evidence of effectiveness across disorders.

**Methods:**

We aimed to comprehensively evaluate its scientific rationale by comparing immediate effects of two weight support levels (20%, 40%) to unsupported (0%) treadmill walking on neural and biomechanical measures in children with unilateral cerebral palsy (CP) and typical development (TD). We hypothesized BWS would demonstrate positive effects only in CP. Participants included 10 with TD and 8 with CP (mean age = 14.6 and 15.4 years, respectively).

**Results:**

Minimal or no group differences or BWS effects were found for synergy number, structure or Walk-DMC, whereas the Gait Deviation Index (GDI) showed a significant interaction with 20% BWS where the dominant side in CP improved with 20% BWS while both sides in TD worsened. Beta band EEG activation from 0% to 20% BWS showed a significant triple interaction increasing in the non-dominant and decreasing in the dominant hemisphere in TD, while increasing in both in CP. A worsening trend was seen with 40% BWS in all measures except z scores.

**Conclusion:**

BWS has beneficial effects on kinematics in CP supporting the basic premise for use in neurorehabilitation at the body structure level.

## 1 Introduction

Cerebral Palsy (CP), which affects approximately 1 in 345 children in the U.S. (1), is a heterogeneous group of neurological disorders due to injuries or insults to the developing brain specifically disrupting motor as well as other aspects of development (2, 3). Walking ability, often a major concern for families of children with CP, may be limited by spasticity, dystonia, poor motor control, muscle weakness, and secondary musculoskeletal changes (4). Mobility levels in CP, as described by the Gross Motor Function Classification System (GMFCS) (5), range from walking independently with only mild incoordination to being dependent on others for all mobility. Many interventions aim to improve mobility in CP to promote greater functional independence and participation in everyday life. Surgical options include tendon lengthening and selective dorsal rhizotomy whereas non-surgical options include targeted muscle chemo denervation, oral anti-spasticity medications, and intensive task-specific physical therapy training, e.g., partial body weight supported treadmill training (6, 7), which is the focus of the current study.

### 1.1 Review of related work

The neurophysiological basis for body weight support (BWS) treadmill training (TT) was established by studies in spinal-lesioned cats, subsequently translated to humans, demonstrating that this type of intervention involving task-oriented training and progressive practice could promote locomotor recovery (8, 9). A harness was required to provide postural support when no longer present or severely impaired and it was further suggested that decreasing the load on the lower limbs would facilitate step initiation during treadmill walking. Despite little scientific evidence to support its efficacy or effectiveness, BWS TT was rapidly adopted by the rehabilitation community to improve mobility in multiple neurological disorders. A large NIH-funded randomized clinical trial, the Locomotor Experience Applied Post-Stroke or LEAPS) Trial was conducted on over 400 individuals 2 or 6 months post-stroke (10). It compared early or later BWS TT to an equal amount of time with a therapist working on functional mobility. Unexpectedly, neither BWS TT group had superior outcomes compared to standard care, leading many to challenge its implementation in neurorehabilitation (11) with the evidence still insufficient to support this in individuals with disorders such as stroke, spinal cord injury and traumatic brain injury (12). While some of the earlier studies in CP, including randomized controlled trials, had also failed to show that BWSTT is superior to overground walking or other effective mobility training approaches (6, 13, 14), a more recent evidence synthesis (7) now lists this among the effective strategies for functional mobility improvement in this population, which is difficult to reconcile given the results in other populations.

In clinical and research settings, the amount of body weight support may range from 0% to 50% (15–17), and this factor alone may contribute to differences in outcomes across studies. Unloading levels have largely been determined through subjective visual inspection of gait patterns that most closely mimic natural walking characteristics (18). Therefore, more rigorous and comprehensive investigations are warranted to elucidate peripheral and central nervous system neuromuscular control mechanisms and biomechanical adaptations across different body weight support levels, thereby providing evidence to establish objective parameters for optimizing BWS protocols in clinical practice.

The present study investigates the effects of BWS during treadmill walking by comparing individuals with unilateral CP to an age-matched cohort with typical development (TD) across three BWS conditions (0%, 20%, and 40%). An analysis of muscle synergies, defined as groups of muscles that are recruited and activated as a single unit (19–23), was utilized as a primary outcome of neural control, including synergy number, Walk-DMC (dynamic motor control index during walking), and quantitative measures of group- and individual-specific synergy structures based on clustering analysis and z-score distributions. Previous research showed that fewer synergies and a lower Walk-DMC index in CP, indicated reduced motor command complexity when compared with typically developing children (24). Distinct differences in muscle synergy structures between CP and typically developing children have also been observed during unsupported treadmill walking, suggesting altered neuromuscular coordination strategies in CP (25, 26). Additionally, while primarily demonstrated in healthy adults, electroencephalographic (EEG) analysis has demonstrated that muscle synergy activation patterns during walking can be successfully decoded from cortical signals (27). Thus, we also included measures of cortical activation patterns in motor-related brain regions using EEG, along with biomechanical outcomes such as temporal-spatial gait parameters and the gait deviation index (GDI) (28).

### 1.2 Hypotheses

Based on the assumption that unweighting improves kinematic patterns in those with gait abnormalities, we anticipated that synergy numbers and Walk-DMC values in CP would increase with BWS along with related improvements in gait parameters, and that their synergy structures and EEG patterns would become more similar to those more commonly seen in TD. In contrast, we anticipated that unweighting was not likely to improve gait in TD, and therefore, outcomes would remain basically unchanged or worsened in that cohort.

## 2 Materials and methods

### 2.1 Participants

The initial group of participants included 9 children with unilateral CP (7 females, 2 males) and 10 children with TD (8 females, 2 males) (Table 1). The greater numbers of females with CP recruited was not by design, since participants of both sexes were welcome to participate. However, to control for any possible differences in these data with respect to sex, although there is no evidence to suspect sex differences except for shorter step lengths in females due to height differences, we recruited controls to match the make-up of the group with CP. Participants ranged in age from 7 to 21 years with a mean of 15.4 years in CP and 14.6 for TD (*p* – 0.61). Height and weight were slightly but not significantly greater in TD (*p* = 0.77 and 0.44, respectively). Of the 9 children with unilateral CP, 5 were GMFCS Level I and 4 were GMFCS Level II (Table 1) which are the two highest out of five mobility levels and indicate that all were able to ambulate independently overground without any mobility aids. This was important because to participate in the study, all had to be able to walk on the treadmill without holding onto the handrails. All participants under 18 and their legal guardians or those over 18 provided informed assent and consent, as appropriate. This protocol was approved by the National Institutes of Health Institutional Review Board (Protocol # 13-CC-0110). Only those who could walk on the treadmill without needing to use arm support were included.

**Table 1:** Participant characteristics for those with cerebral palsy (CP) and typical development (TD).

Group ID	Age (yrs)	Height (cm)	Weight (kg)	Handedness	Gender	GMFCS
CP1	14	162	43.7	Right	Female	I
CP2	21	166	54.0	Left	Female	II
CP3	16	180	89.2	Left	Male	I
CP4	17	161	75.7	Right	Female	I
CP5	17	178	63.3	Right	Male	I
CP6	13	156	51.1	Left	Female	II
CP7	17	156	57.2	Left	Female	I
CP8	7	120	18	Right	Female	II
CP9	17	174	82.9	Left	Female	II
**CP mean**	**15.44**	**161.44**	**59**.**46**	–	–	–
TD1	14	167	81.6	Right	Female	–
TD2	16	165	56.4	Right	Female	–
TD3	18	166	62.8	Right	Female	–
TD4	16	171	92.4	Right	Male	–
TD5	14	154	50.5	Right	Female	–
TD6	18	177	100.4	Right	Female	–
TD7	15	164	65.9	Right	Female	–
TD8	13	168	63.9	Right	Female	–
TD9	7	123	20.8	Right	Female	–
TD10	15	183	81.1	Right	Male	–
**TD mean**	**14.6**	**163.8**	**67**.**6**	–	–	–
*p* value	0.61	0.77	0.44			

Bold indicates *p* < 0.05.

### 2.2 Procedures

The experimental protocol consisted of three treadmill walking conditions (0%, 20%, and 40% BWS). Participants initially were instructed to stand still for 2 min to obtain a resting baseline. Then participants walked for 5 min first at a self-selected speed with 0%, then 20% and 40% BWS in a randomized order using the Zero-G harness which maintains a consistent level of unweighting regardless of the vertical position of the center of mass. If a participant was not able to maintain their walking pace or needed to use arm support during a walking condition, the trial was discontinued and recorded as missing data.

Neural and biomechanical measures were collected using three synchronized measurement systems (EEG, EMG, and motion analysis). A 64-channel, wireless, active EEG system (Brain Products, Morrisville NJ) was positioned within a snugly fitted EEG cap on the participant's head and electrodes were placed according to the International 10/20 system with data collected at 1,000 Hz. EMG data were recorded wirelessly (Trigno Wireless, Delsys, Boston, MA, United States) at 1,000 Hz from surface electrodes positioned on the skin over the right and left eight muscle bellies of the tibialis anterior (TA), medial gastrocnemius (MG), soleus (SOL), peroneus longus (PL), rectus femoris (RF), vastus lateralis (VL), medial hamstrings (MH), and hallicus longus (HL). A 10-camera motion capture system (Vicon, Lake Forest, CA) collected kinematic data at 100 Hz. Reflective markers were placed on anatomical landmarks to track the position of the feet, shank, thigh, and pelvis segments. All kinematic data were processed using Visual3D software (C-Motion, Germantown, MD, USA). These data were utilized to compute temporal-spatial gait parameters (i.e., gait speed, cadence and step distance) and to calculate the Gait Deviation Index (GDI). The GDI is a single number that is a validated indicator of the overall degree of gait pathology compared to a normative reference group based on 15 selected kinematic features, as detailed in (28). A GDI score of 100 indicates normal gait with each 10-point increment below that representing one standard deviation from normal. In this study, we utilized gait data from our 10 participants with TD to serve as the reference group.

EMG data were processed using 35 Hz high-pass and 5 Hz low-pass Butterworth filters (6th order). For synergy analysis, EMG data were divided into 20-cycle windows with a one-cycle sliding window and normalized to maximum activation within each window, as previously described (29). Non-negative Matrix Factorization (NMF) was applied to extract muscle synergies from the processed EMG matrices (EMGo) (30): $\mathrm{EM}G_{0}=\overset{n}{\underset{i=1}{\sum }}\;W_{i}C_{i}\;+\;e,\;\mathrm{EM}G_{r}=\overset{n}{\underset{i=1}{\sum }}\;W_{i}C_{i}$ where *n* is the number of synergies from 1 to 16, i is an identification number of each synergy, W represents synergy structure weight vector, *C* indicates activation coefficients, and e is residual error. EMGr is a reconstructed EMG matrix resulting from the multiplication of *W* and *C*.

The number of synergies was determined using a 90% variability accounted for (VAF) threshold as follows: $\;\mathrm{VAF}=1-\;(\mathrm{EM}G_{o}-\mathrm{EM}G_{r}{)}^{2}/\mathrm{EM}G_{o}^{2}$. Based on VAF, the dynamic motor control index during walking (Walk-DMC) was computed to quantify neuromuscular control complexity (24): $\mathrm{walk}-\mathrm{DMC}=100*10\left[\frac{\mathrm{VA}F_{\mathrm{AVE}}-\mathrm{VA}F_{1}}{\mathrm{VA}F_{\mathrm{STD}}}\right]$, where VAF1 represents the VAF with one synergy, and VAFAVE and VAFSTD are the mean and standard deviation of VAF1 across the typically developing cohort. Higher VAF1 resulting in lower Walk-DMC indicates simplified neuromuscular control.

To identify group-specific characteristics under different BWS conditions, muscle synergy structures were classified using k-means clustering and discriminant analyses as detailed in (29). Then, clusters were categorized as CP-specific (C), TD-specific (T), or non-specific based on two-proportion z-test results (*z* > 1.96, *z* < −1.96, or −1.96 ≤ *z* ≤ 1.96, respectively). Within each category, clusters were numbered according to their z-values (e.g., C1 representing the CP-specific cluster with highest *z*-value). To quantitatively assess how much each participant exhibited group-specific muscle synergies, individual weight-averaged *z* scores were computed from the number of synergies and corresponding cluster *z*-values, with positive scores indicating stronger CP representation and negative scores indicating stronger TD representation. This analysis was performed separately for each walking condition and averaged across conditions.

### 2.3 EEG processing

**Figure 1:**
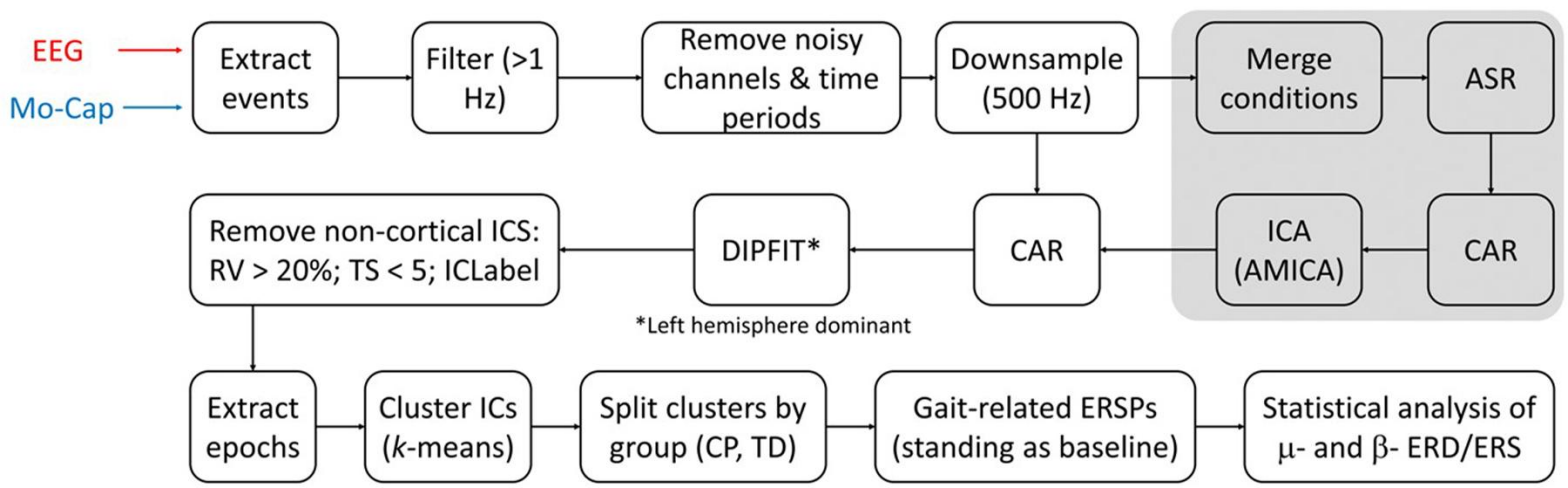
Processing steps for analyzing the differences in motor-related cortical activity between groups (typical development [TD], cerebral palsy [CP]) and walking conditions (with and without 20% BWS) from scalp-recorded EEG.

EEG data analysis procedures were based on our prior studies (25, 31) and are summarized in Figure 1. EEG and motion capture data were synchronized to align gait events with EEG data. EEG data were then high pass filtered at 1 Hz and noisy channels and time periods were removed before downsampling and then concatenating across conditions (standing, walking with and without 20% BWS). Next, an artifact subspace reconstruction (ASR) algorithm (32) was used to identify and reconstruct time periods corrupted by non-stereotypical artifact. The ASR-cleaned data were then common average referenced and an adaptive mixture independent component analysis (AMICA) (33) was used to extract independent sources (ICs) from EEG. The AMICA transformation was then applied to the downsampled, non-ASR cleaned data sets. Next, equivalent dipoles were fit to each IC using the DIPFIT toolbox in EEGLAB with a template, 3 shell boundary element head model. ICs with dipole fits having greater than 20% residual variance (RV) (31) and topographical sparseness (TS) less than 5 (34) were rejected. IC power spectra, scalp topography, and dipole location were also visually inspected to remove non-cortical sources such as eye blink, EMG, etc. Dipole locations were then adjusted based on hand dominance, such that the left hemisphere (right hand) was represented as the dominant side for all participants.

Next, individual strides from consecutive heel-strikes were extracted as were non-overlapping 2 s epochs from the standing baseline data. K-means was then used for clustering to pool ICs from both groups across all conditions using their dipole coordinates, power spectral density (PSD), and inter-trial coherence (ITC) as coordinates in the parameter space, where PSD and ITC were reduced to 3 dimensions using principal component analysis (PCA). PSD and ITC were equally weighted in the k-means algorithm while the dipole coordinates were assigned a weight three times greater. Two sensorimotor-related clusters were identified, one in each hemisphere, based on the centroid location and confirmed by the presence of mu and beta event-related desynchronization (ERD) during walking relative to standing. The event related spectral perturbations (ESRPs) of each gait cycle were computed to quantify the de-synchronization or synchronization at each time point, by dividing the walking power spectra by the respective standing (rest) mean spectra for each IC. The ICs within each cluster were split by group (TD, CP) and the overall group mean ERSP for each condition was computed. Finally, statistically significant differences in the mean mu (8–13 Hz) and beta (15–30 Hz) ERSPs for each cluster were computed across conditions and groups using nonparametric bootstrapping via the condstat function in the EEGLAB using 2,000 points of surrogate data with the significance (alpha) set to 0.05.

### 2.4 Statistical analysis

Statistical analyses for synergy number, *z* scores, temporal-spatial and kinematic gait data, and EEG ERD/ERS values were performed using a general linear mixed model in SPSS (version 31.0 using the full factorial default model with polynomial contrasts) with group (CP or TD) as the between subject variable and BWS as the within subject variable, first with all who had complete data for 0% and 20% BWS and then for all who had complete data for 0, 20% and 40% BWS. The statistical significance was set at *p* < 0.05. *Post hoc* tests were performed as indicated. Baseline data as well as change scores were correlated across outcome categories using Pearson *r* for the entire sample and those with CP separately.

## 3 Results

### 3.1 Participants

While data from all participants and conditions were used to determine the clusters, statistical comparisons of conditions, limbs and groups only included participants able to perform one or both BWS conditions. Of the 9 with CP and 10 with TD who were able to perform the no BWS conditions, one participant (CP8) could not perform either the 20% or 40% BWS condition without holding onto the handrails; therefore, was only included in the clustering analysis. Additionally, two participants with CP (CP3 and CP4) were not able to complete the 40% BWS condition for the full duration without holding the handrails. For two other participants with CP (CP1 and CP2) and two with TD (TD1 and TD10), the BWS harness moved vertically up and down during the 40% condition, resulting in discomfort and inaccurate motion capture data; therefore, these participants were excluded from the 40% BWS analysis. Finally, a technical error in the synchronization signal between EMG, EEG and motion capture occurred during 40% BWS condition in TD2 who was excluded as well. To test the assumption that those who could not perform or were excluded from the highest BWS support condition were less functional due to age or other factors, we compared participants who could not perform the 40% BWS condition with those who could. Interestingly, age and baseline temporal-spatial or GDI data did not differ significantly between these subgroups; synergy numbers also did not differ (see Table 2 a & b; all *p* values > 0.80). The distribution of GMFCS levels in CP was similar in those who could and could not perform the 40% BWS condition.

**Table 2:** Mean outcomes for groups with typical development (TD) and cerebral palsy (CP) for each body weight support (BWS) condition.

Outcomes	*n* for TD/CP	TD	CP
Synergy number			
Baseline	10/8	5.30 (0.48)	5.25 (0.46)
20% BWS	10/8	5.20 (0.63)	5.25 (0.46)
40% BWS	7/4	5.00 (0.00)	4.75 (0.50)
Walk-DMC			
Baseline	10/8	100.0 (10.0)	93.1 (9.61)
20% BWS	10/8	100.0 (10.0)	98.4 (14.82)
40%BWS	7/4	100.0 (10.0)	94.2 (8,04)
Synergy *z* score			
Baseline	10/8	−16.4 (5.68)	12.0 (11.25)
20% BWS	10/8	−18.9 (7.63)	12.0 (10.51)
40%BWS	7/4	−14.3 (6.12)	13.5 (7.64)
Gait speed (m/s)			
Baseline	10/8	0.99 (0.11)	0.89 (0.10)
20% BWS	10/8	0.99 (0.11)	0.89 (0.10)
40%BWS	7/4	0.99 (0.11)	0.89 (0.11)
Cadence			
Baseline	10/8	104.3 (5.52)	102.4 (10.7)
20% BWS	10/8	102.7 (5.70)	101.4 (10.5)
40%BWS	7/4	100.1 (7.2)	96.3 (10.6)
Dominant step distance			
Baseline	10/8	0.51 (0.05)	0.50 (0,03)
20% BWS	10/8	0.51 (0.06)	0.49 (0.03)
40%BWS	7/4	0.51 (0.08)	0.47 (0.09)
Non-dominant step distance			
Baseline	10/8	0.50 (0.07)	0.48 (0.04)
20% BWS	10/8	0.51 (0.07)	0.49 (0.05)
40%BWS	7/4	0.51 (0.09)	0.48 (0.05)
Gait deviation index - dominant			
Baseline	10/8	95.7 (10.8)	77.8 (10.8)
20% BWS	10/8	91.0 (10.5)	82.0 (10.0)
Gait deviation index – non-dominant			
Baseline	10/8	95.8 (8.40)	72.4 (10.8)
20% BWS	10/8	91.9 (8.83)	73.4 (6.26)
Mu ERD – dominant			
Baseline	9/7	−4.76 (1.52)	−4.79 (3.45)
20% BWS	9/7	−4.61 (1.24)	−5.36 (4.14)
Mu ERD – non-dominant			
Baseline	8/5	−3.64 (1.29)	−4.84 (1.36)
20% BWS	8/5	−3.93 (1.56)	−5.38 (2.28)
Beta ERD – dominant			
Baseline	9/7	−2.96 (0.78)	−3.26 (1.13)
20% BWS	9/7	−2.84 (0.64)	−3.64 (1.00)
Beta ERD – non-dominant			
Baseline	8/5	−2.70 (0.64)	−3.10 (0.33)
20% BWS	8/5	−3.03 (0.30)	−3.19 (0.72)
*Z* score			
Mean	10/8	−16.4 (4.5)	12.1 (9.5)
Baseline	10/8	−17.4 (5.2)	11.0 (10.2)
20% BWS	10/8	−17.0 (6.0)	13.3 (8.8)
40% BWS	7/4	−14.6 (6.1)	12.5 (7.4)

Note that values for 40% BWS are not directly comparable to the larger sample for 0 and 20% BWS.

### 3.2 Muscle synergy results

Across all conditions, the mean number of gait cycles used for the analyses was 216.1 ± 35.5 for the group with TD and 214.5 ± 46.9 for the group with CP which did not differ across groups (*p* = 0.91). The GLM analysis revealed no significant group differences in synergy number in any condition and no interactions for the 0% and 20% BWS (BWS condition *p* = 0.71; group *p* = 0.71; group by condition interaction *p* = 1.00) and 0, 20% and 40% BWS cohorts (BWS condition *p* = 0.10; group *p* = 0.80; interaction *p* = 0.82), indicating that the group with CP did not demonstrate baseline differences in synergy number, nor did BWS significantly alter synergy numbers in either group (see Table 2 for values of all measures by group and condition).

While the mean walk-DMC for the group with CP was lower than 100, the GLM results comparing the 0% and 20% BWS conditions did not demonstrate any significant condition or group differences or interactions (*p* = 0.19 for BWS, *p* = 0.19 for interaction, *p* = 0.40 for group). Interestingly, there was a significant difference (worsening) for the BWS condition (*p* = 0.04) when comparing 20% and 40% in the whole sample (*p* = 0.41 for interaction, *p* = 0.97 for group). The mean value for the group with CP moved closer to the normative TD Walk-DMC value of 100 with 20% BWS but further away with 40% BWS.

#### 3.2.1 Clustering: muscle synergy

**Figure 2:**
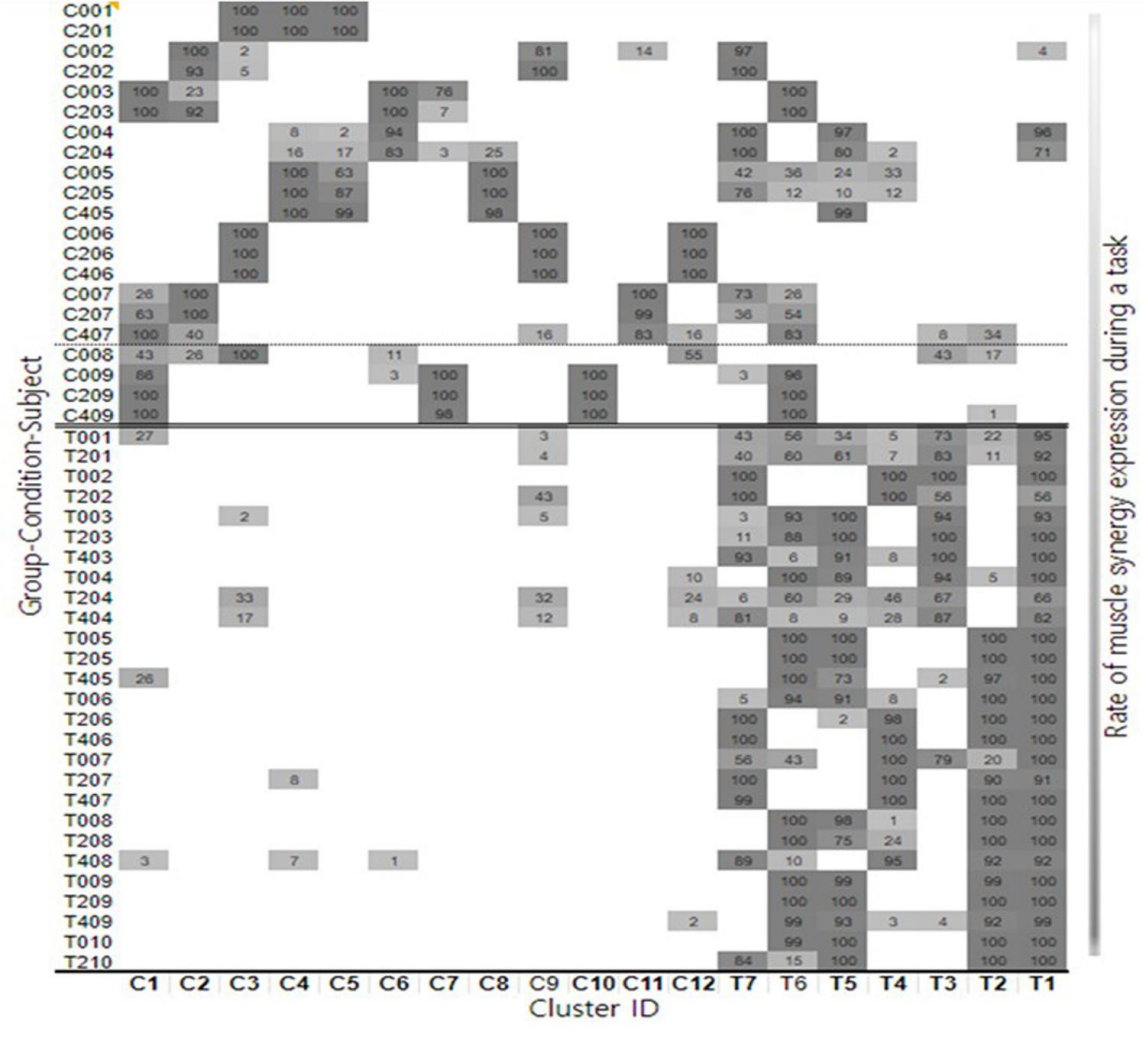
Summary for each participant and cluster showing the distribution of individual synergy structures and how these change with body weight support conditions.

Nineteen distinct clusters across all participants and conditions were identified. Muscle activation patterns for each cluster are shown in Supplementary Figure S1. To determine whether a cluster was specific to the TD or CP group, the proportion of synergies for each cluster was calculated using the two-proportion z-test. Based on these values, 12 CP-specific clusters and 7 TD-specific clusters were identified (Figure 2). Of the 12 CP clusters, 6 were present in both groups (C1, 3, 4, 6, 9, 12) whereas the rest were present only in CP (C2, 5, 7, 8, 10, 11). Clusters C8, C10, and C11 were predominantly observed in participants CP5, CP9, and CP7, respectively, indicating that these clusters may be subject specific. Of the 7 TD clusters, all were present in both groups and there were no TD clusters that were subject specific.

#### 3.2.2 Muscle synergy structure changes with BWS

With increasing BWS, 21.9% of synergies showed changes in TD and 11.5% in CP with most reflecting reduced activation in the Tibialis Anterior and Extensor Hallucis Longus muscles during weight acceptance. We then quantified changes in synergies within clusters without and with BWS for each participant by calculating a weight-averaged *z* score for each condition (29). Similar to the cluster *z* score, a greater positive shift in the individual weight-averaged *z* score with an increase in BWS indicated that a participant's synergies were becoming more CP-specific, and a greater negative shift signified that these were becoming more TD-specific. The mean shift from 0% to 20% BWS was minimal but in the direction of CP for both groups, with a slight mean shift in the direction of TD from 20% to 40% for those able to do that condition. *Z* scores are summarized in Table 2 by group and condition. We also calculated a mean z score across all conditions. All differed between groups as expected (*p* < 0.001). Yet, there was no significant main effect for BWS conditions for the 0, 20% or for the 0, 20% and 40% BWS cohorts (*p* = 0.29 and 0.67, respectively) or interaction (*p* = 0.43 and 0.38, respectively).

### 3.3 Gait analysis results

#### 3.3.1 Temporal-spatial data

There were no significant group differences at baseline (cadence *p* = 0.66, speed *p* = 0.08, dominant step distance *p* = 0.61, non-dominant step distance *p* = 0.33: *n* = 8 CP, 10 TD). There were no appreciable or significant changes in gait speed, likely because the treadmill was set at each participant's freely selected baseline speed across conditions (Table 3). However, there were some small but significant changes in cadence which decreased slightly in both groups with 20% BWS (Table 3). Cadence also decreased in both groups from 20% to 40% BWS (Table 5). For step distance, there was a slight increase on the dominant side and a larger increase in both groups on the non-dominant side with increased BWS (Table 4). Speed showed only a 0.01 m but significant decrease in the group with TD with 40% BWS.

**Table 3:** General linear mixed model results for the temporal spatial gait measures from the baseline and 20% BWS conditions (bold = *p* < 0.05).

Effects	Cadence	Step distance	Speed
BWS condition	**0.02**	0.26	0.33
Condition X group	0.57	0.31	0.23
Limb	–	0.44	–
Limb X group	–	0.66	–
Condition X limb	–	**0**.**02**	–
Group (between)	0.69	0.38	0.23-
Cond X limb X group	–	0.08	

**Table 4:** Condition, group, and limb effects for cadence, step distance, speed.

Effects	Cadence	Step distance	Speed
Condition	**0** **.** **03**	**0**.**054**	**0**.**04**
Condition X group	0.79	0.62	0.22
Limb		0.85	
Limb X group		0.92	
Condition X limb		0.33	
Condition X limb X group		0.60	
Group	0.46	0.41	0.08

Bold indicates *p* < 0.05.

**Table 5:** General linear mixed model results for the temporal spatial gait measures from the 20% to 40% BWS conditions (bold = *p* < 0.05).

Effects	Cadence
BWS condition	0.68
Condition X group	0.06
Limb	0.23
Limb X group	0.16
Condition X limb	0.27
Cond X limb X group	0.07
Group	**0** **.** **001**

#### 3.3.2 GDI values

**Figure 3:**
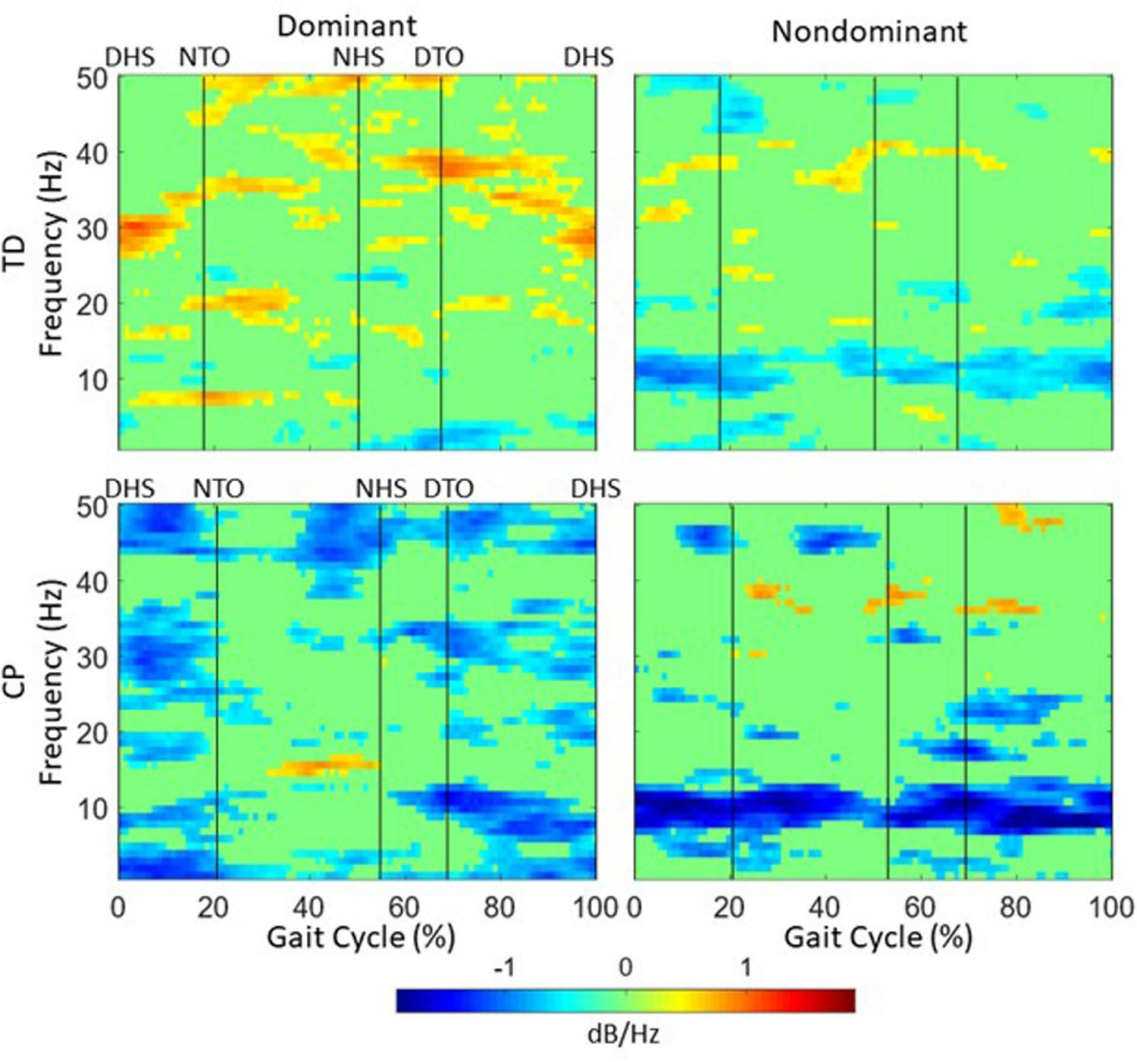
Mean GDI values for the groups with typical development (TD) or cerebral palsy (CP) for the 0% and 20% body weight support (BWS) conditions.

GDI values could only be calculated for baseline and 20% BWS because there was a frequent loss of markers in the 40% BWS condition. The groups had significantly different GDI values, with the group with CP tending to improve on the dominant side only while the group with TD had marginally lower scores on both sides, with interaction *p* values close to but not reaching significance (Table 2; Figure 3).

### 3.4 EEG results

**Figure 4:**
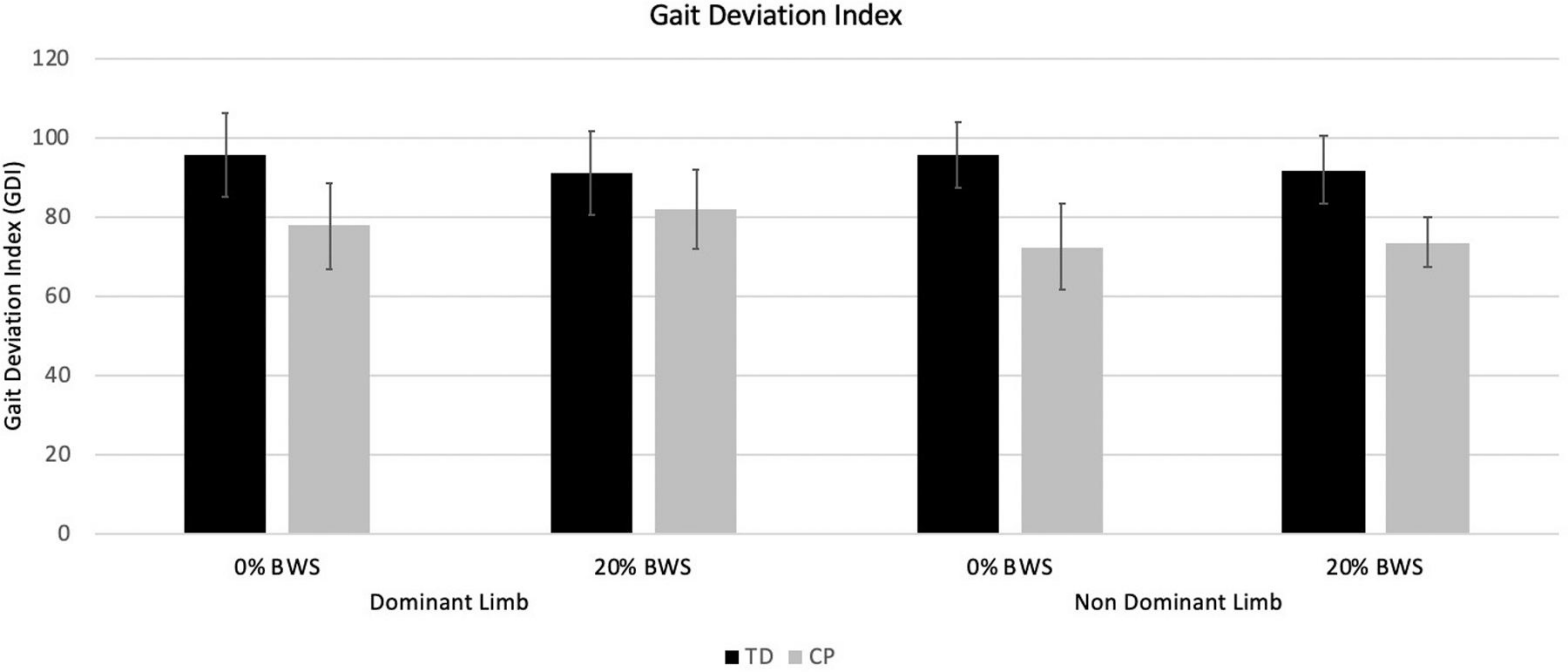
Relative ERD/ERS over the gait cycle between the 20% BWS and no BWS walking for TD (top) and CP (bottom) groups in the dominant (left) and nondominant (right) sensorimotor clusters. Negative values indicate larger ERD in 20% BWS. Plots are masked for significance (*p* < 0.05) with nonsignificant values set to 0 dB.

We computed ERD data for alpha (mu) and beta frequencies in the non-dominant and dominant motor clusters. Not all participants had an independent component in one or both clusters (a total of 9 TD and 5 CP had cortical sources in the non-dominant motor cluster, and a total of 9 TD and 7 CP had sources in the dominant motor cluster; 8 TD and 5 CP had sources in both hemispheres). Both TD and CP showed significant desynchronization during baseline and 20% BWS walking in both hemispheres compared to standing, indicating increased cortical activity. Comparing between 20% BWS and no BWS, there were differential effects over the gait cycle between groups whereby relative desynchronization in mu band was present in the nondominant but not dominant hemisphere in TD (Figure 3). In CP, relative mu and beta desynchronization in 20% BWS was present both hemispheres and was stronger on the nondominant side (Figure 4).

#### 3.4.1 GLM results for EEG during BWS by group

The only significant result was a triple interaction (group X hemisphere X condition) in the beta band (Table 6). This was explained by differential effects of BWS by hemisphere across groups (Table 2) wherein the largest increase in beta ERD with BWS was in the dominant hemisphere in CP and the nondominant hemisphere in TD whereas there was a slight decrease for TD and slight increase for CP in the dominant and nondominant hemispheres, respectively.

**Table 6:** General linear mixed model results for the EEG event-related desynchronization (ERD) measures for the alpha or mu and beta frequency bands from the 0 to 20% BWS walking conditions (bold = *p* < 0.05).

Effects	Mu ERD	Beta ERD
BWS condition	0.15	0.30
Condition X group	0.25	0.67
Hemisphere	0.53	0.51
Hemisphere X group	0.50	0.61
Condition X hemisphere	0.60	0.64
Condition X hemisphere X group	0.50	**0** **.** **048**
Group	0.44	0.18

### 3.5 Correlations between neural and biomechanical measures

The baseline correlations in the entire sample showed several relationships across neural and biomechanical measures with the relationship between the synergy *z* score and the GDI the most notable with more TD-like scores related to more TD-like kinematics. When the same measures were correlated within groups, only the relationships between Beta ERD and gait speed remained for CP (r = 0.84; *p* = 0.04; Table 7).

**Table 7:** Significant correlation and associated *p*-values between baseline neural and biomechanical measures.

Neural measures	Gait speed	GDI dominant	GDI non-dominant	Dominant step distance
Z score		−0.60 (<0.01)	−0.69 (<0.01)	
Synergy number				0.47 (0.04)
Beta ERD dominant	0.58 (0.02)			

## 4 Discussion

The main question addressed here was whether body weight support had a positive effect on neural and/or biomechanical measures during gait in children with unilateral CP. All participants with CP were high-functioning, able to walk independently on a treadmill with mean baseline gait speed comparable to the TD group. Neural outcome measures included muscle synergy analyses (number of synergies, walk-DMC values, and *z*-scores) and motor related cortical activation in bilateral sensorimotor regions during 0% and 20% BWS conditions. Biomechanical measures included temporal-spatial gait parameters and the Gait Deviation Index. Baseline neural measures were also correlated with biomechanical measures to examine interrelationships across variables.

For the sake of comparison across conditions, participants were not allowed to hold onto the treadmill rails, which affected protocol completion rates: one participant with CP could not perform any BWS condition, while four with CP and three with TD could not complete the 40% BWS condition. Interestingly, participant characteristics (age and GMFCS levels) and baseline measures (gait data and synergy numbers) did not differ between those who could and could not complete this condition.

### 4.1 Muscle synergy number or walk-DMC

Muscle synergy numbers and Walk-DMC are established measures of motor control complexity, with children with CP typically utilizing fewer synergies, indicating simplified control strategies (24). However, our high-functioning CP cohort here showed no significant differences in synergy numbers between groups. This finding aligns with recent research in similar functional populations (35). Additionally, while Walk-DMC scores were lower in the CP group as expected, this measure did not significantly differentiate between groups at baseline or in response to BWS. The modifiability of muscle synergies through intervention remains an open question (36). Previous intervention studies have shown inconsistent effects on synergy numbers despite improvements in kinematic outcomes (37). Our finding of unchanged synergy numbers with BWS was similar to that of Chen et al. (43); however, unlike in our study, they found differences in synergy activation patterns, recommending these as a more sensitive measure.

### 4.2 Synergy structures

Analysis of group-specific z scores revealed distinct muscle synergy structures between groups that remained relatively stable across BWS conditions (ranging from 11 to 13.3 in CP and from −17.4 to −14.6 in TD), confirming their distinct control strategies. This stability in muscle synergy structures suggests that temporary BWS may not substantially alter established neuromuscular control strategies, and suggests that these coordination patterns, once established, may remain relatively fixed despite transient mechanical modifications (36). This view is consistent with (38) who stated that muscle synergies are encoded prior to the onset of walking (38) and may therefore not be modifiable; However (37), which was the first study to report on changes in synergy activations in response to interventions using a similarity score based on a sample of age-matched children with TD showed a significant change in the direction of TD for those who underwent selective dorsal rhizotomy, but not for those who had orthopaedic surgery or toxin injections. Similarly, a previous study by our group demonstrated worsening synergy patterns with another environmental modification, i.e., walking on a narrower path (26) also providing a strong counter argument along with Chen et al. (43).

### 4.3 Gait outcomes

The basic premise for the use of BWS in CP and other neurorehabilitation populations is to improve and repetitively practice kinematic patterns, so perhaps it is no surprise that with the minimal functional impairment in our cohort with unilateral CP, the GDI values were significantly different between groups at baseline (0% BWS) and responded differentially to BWS. As presumed, the mean GDI improved in CP on both sides although the change was larger and only significant on the dominant side, whereas this tended to worsen on both sides in the group with TD. One possible explanation for the difference in the magnitude of response across legs in CP may be that the neural control in the dominant leg was more flexible and adaptable, vs. more constrained on the non-dominant side. Interestingly, the GDI regressed from 20% to 40% suggesting that 40% was not optimal for these participants. However, it may be the case, in contrast to this sample, that participants with CP who have greater weakness, spasticity, and functional impairments (i.e., GMFCS higher than Level II) would benefit from greater weight support. Cadence decreased significantly from 0% to 20% BWS in both groups by the same amount. From 20% to 40%, cadence again significantly decreased and speed increased but almost imperceptibly.

### 4.4 EEG outcomes

Although all EEG desynchronization values were consistently higher in the group with CP, suggesting greater cortical activation, these were not significantly different between groups at baseline. Participants in this study were a subset of those from a slightly larger group with CP who did treadmill walking with no BWS and had significantly greater EEG activation than the group with TD (25), suggesting that the finding of no significant difference here was related to the smaller sample size. EEG activation changes from 0% to 20% BWS did show a significant triple interaction between BWS X hemisphere X group in the beta band. On average, for the TD group, brain activation tended to increase in the non-dominant hemisphere and decrease in the dominant hemisphere with increasing BWS, whereas in the group with CP activation increased in both hemispheres. Increased brain activation with task difficulty often indicates greater attention or effort was needed, which in the group with TD was mostly seen to be focused on the non-dominant hemisphere which controls their non-dominant leg. In those with unilateral CP, bilateral activation is more commonly observed due to retention of ipsilateral pathways and brain reorganization post-injury (39).

### 4.5 Correlations among neural and biomechanical measures

When evaluating interrelationships across neural and biomechanical outcomes, *z* scores showed moderate inverse correlations with the dominant and non-dominant GDI, indicating that synergy structures more similar to TD were associated with better kinematics. Beta ERD in the dominant hemisphere was moderately correlated with gait speed in the group as a whole. This was the only significant relationship retained in the within group correlations where the group with CP showed strong correlations between Beta ERD on the dominant side and gait speed. EEG data were interestingly not correlated with synergy data, even though both represent aspects of cortical control of movement. The stronger relationship of synergy structures with kinematics and their lack of a correlation with brain activation raises the question of their direct cortical control (40). This contrasts with (27) who found that movement-related slow cortical potentials (0.5–10 Hz) were linked to synergy structures, which may differ from findings in the alpha and beta bands which we investigated here How direct the link between the brain and the muscle synergies is, however, was challenged by (41) who evaluated whether spinal motor neurons, even more closely linked to cortical output, demonstrated synergies during a complex upper limb task. The motor neuron synergies they identified better discriminated individual finger forces than muscle synergies and, in a few cases, motor neurons innervating a given muscle were active in separate synergies. This indicates that cortical mapping onto muscles is not direct but involves other even closer levels of the nervous system in dimensionality reduction.

### 4.6 Clinical and research implications

Since stepping is essentially reflexive in spinal-lesioned animals, electrophysiological methods have been used to examine neural pathways and reflexes in response to body weight unloading (44) with one study showing that cutaneous reflexes were enhanced with unloading (45) with potentially positive effects on motor output (46). It is theorized that BWS during treadmill training supplies the injured nervous system with necessary and appropriate sensory input signals for stimulating intact spinal cord networks, most notably central pattern generators (CPG), which can be directed towards improving the control of walking (11, 47). BWS treadmill training aimed to utilize spinal reflexes and networks to facilitate stepping originally for patients with complete spinal cord injuries. Cortical involvement during walking based on task-specific EEG studies in healthy adults has been shown to be greater than previously assumed and greater engagement of brain activity during motor training may enhance effectiveness (31, 42), Thus, gait rehabilitation is more complex in disorders such as stroke and CP where cortical input is present but may be abnormal and reciprocal stepping is present but muscle activation patterns and joint motions are not as efficient, smooth or forceful as seen in those without these disorders. Weakness and poor postural control may further limit gait rehabilitation in CP and stroke but are accommodated rather than remediated by harnessed or device-provided weight support; and therefore, often need to be addressed separately.

As anticipated, the effects of BWS on muscle, brain, and gait measures in those with TD were negligible and even somewhat negative in some cases (e.g., in their GDI values which in contrast improved in CP). Gait in those with typical development is optimized for efficiency and low energy expenditure and perturbations such as reducing body weight are unlikely and unnecessary to improve mobility function. Increases in brain activation with increased BWS was also only seen in CP indicating that this required greater attention or effort in those with mobility challenges, with a negligible effect in TD likely due to the greater flexibility and adaptability of their motor control system. This study demonstrated that providing some level of BWS produces greater biomechanical similarity to gait in those with TD and thus may offer some training benefits that should be combined with strengthening and exercises to enhance postural control. However, the relationship between the level of BWS and improved clinical, neural and/or biomechanical outcomes in CP may not be entirely linear as it appears when going from 0% to 20% BWS and instead may be more curved or even an inverted-U shape as shown by improvements plateauing or even reversing in some cases with 40% BWS. From a clinical standpoint, it seems important to advise that the level be determined carefully using objective parameters when available so as to maximize the training benefit for each individual. Perhaps other more invasive interventions such as orthopaedic and neurosurgery or botulinum toxin injections that also address biomechanical and/or neural impairments common in CP will be needed for larger effects. These interventions must ultimately demonstrate that they significantly improve function or participation in children with CP to justify their use, either alone or in combination, in clinical practice.

### 4.7 Limitations

The major limitation here was the small sample size of those who could perform the baseline condition (0% BWS) and one or both BWS conditions (20% and 40% BWS). The study design also required that those with CP have a high level of mobility which limited the ability to detect group differences when compared to the TD group, given the similar functional walking abilities of both groups. However, several significant immediate effects of BWS on neural and biomechanical measures were identified that in some cases were similar and other cases divergent across groups. Finally, this was not a training study, so longer-term effects of BWS on these outcomes were not addressed here.

### 4.8 Conclusion

BWS can alter joint kinematics in CP in the direction of greater similarity to those without CP which were also related to synergy z scores, likely because the timing and magnitude of muscle activation produces joint motion patterns. These findings suggest potential clinical benefits from BWS for those with CP. EEG did relate to gait speed as a measure of function but did not vary with muscle synergies that many propose are encoded or controlled by the CNS, thus continuing the debate on the role of muscle synergies in the neural control of movement (40).

## Data Availability

The raw data supporting the conclusions of this article will be made available by the authors, without undue reservation.

## Author's note

The contributions of the NIH author(s) are considered Works of the United States Government. The findings and conclusions presented in this paper are those of the author(s) and do not necessarily reflect the views of the NIH or the U.S. Department of Health and Human Services.

## Supplementary material

Supplementary Figure S1 Muscle synergy activation clusters with the timing of the cluster's muscle activation across the gait cycle shown in the top left corner of each figure, peak activation time in the gait cycle (top right) and structures (bottom). The left column consists of clusters specialized for cerebral palsy, and the right one is for typical development. Subfigures are arranged based on peak activation time from top to bottom for each column. Data are expressed as mean and standard deviation. The labels of bar plots are combined with leg side and muscle names as follows: D, dominant leg; N, non-dominant leg; TA, tibialis anterior; EH, extensor hallucis longus; LG, lateral gastrocnemius; SO, soleus; RF, rectus femoris; VL, vastus lateralis; ST, semitendinosus; BF, biceps femoris.
