# Supplementary material for: Does body weight support improve neural and biomechanical measures during treadmill gait in children with unilateral cerebral palsy?
Source: Front Rehabil Sci. 2025 Oct 23;6:1607515. doi: 10.3389/fresc.2025.1607515 (PMC12588987; doi:10.3389/fresc.2025.1607515)
Supplement: Supplementary file 1 [file Supplementaryfile1.docx]

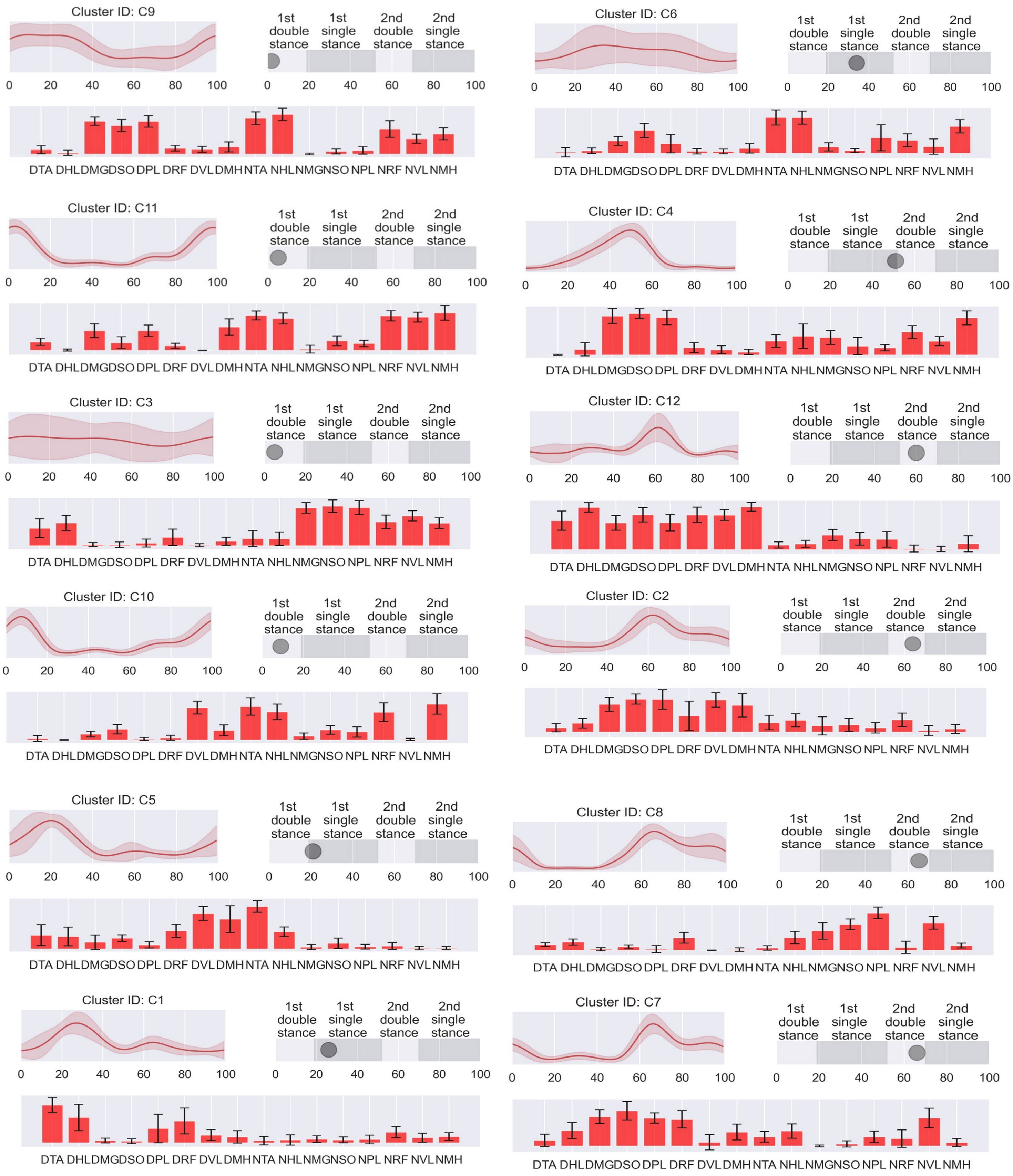
Cluster ID: C9
1st double stance
1st single stance
2nd double stance
2nd single stance
0 20 40 60 80 100
DTA DHL DMG DSO DPL DRF DVL DMH NTA NHL NMG NSO NPL NRF NVL NMH
Cluster ID: C6
1st double stance
1st single stance
2nd double stance
2nd single stance
0 20 40 60 80 100
DTA DHL DMG DSO DPL DRF DVL DMH NTA NHL NMG NSO NPL NRF NVL NMH
Cluster ID: C11
1st double stance
1st single stance
2nd double stance
2nd single stance
0 20 40 60 80 100
DTA DHL DMG DSO DPL DRF DVL DMH NTA NHL NMG NSO NPL NRF NVL NMH
Cluster ID: C4
1st double stance
1st single stance
2nd double stance
2nd single stance
0 20 40 60 80 100
DTA DHL DMG DSO DPL DRF DVL DMH NTA NHL NMG NSO NPL NRF NVL NMH
Cluster ID: C3
1st double stance
1st single stance
2nd double stance
2nd single stance
0 20 40 60 80 100
DTA DHL DMG DSO DPL DRF DVL DMH NTA NHL NMG NSO NPL NRF NVL NMH
Cluster ID: C12
1st double stance
1st single stance
2nd double stance
2nd single stance
0 20 40 60 80 100
DTA DHL DMG DSO DPL DRF DVL DMH NTA NHL NMG NSO NPL NRF NVL NMH
Cluster ID: C10
1st double stance
1st single stance
2nd double stance
2nd single stance
0 20 40 60 80 100
DTA DHL DMG DSO DPL DRF DVL DMH NTA NHL NMG NSO NPL NRF NVL NMH
Cluster ID: C2
1st double stance
1st single stance
2nd double stance
2nd single stance
0 20 40 60 80 100
DTA DHL DMG DSO DPL DRF DVL DMH NTA NHL NMG NSO NPL NRF NVL NMH
Cluster ID: C5
1st double stance
1st single stance
2nd double stance
2nd single stance
0 20 40 60 80 100
DTA DHL DMG DSO DPL DRF DVL DMH NTA NHL NMG NSO NPL NRF NVL NMH
Cluster ID: C8
1st double stance
1st single stance
2nd double stance
2nd single stance
0 20 40 60 80 100
DTA DHL DMG DSO DPL DRF DVL DMH NTA NHL NMG NSO NPL NRF NVL NMH
Cluster ID: C1
1st double stance
1st single stance
2nd double stance
2nd single stance
0 20 40 60 80 100
DTA DHL DMG DSO DPL DRF DVL DMH NTA NHL NMG NSO NPL NRF NVL NMH
Cluster ID: C7
1st double stance
1st single stance
2nd double stance
2nd single stance
0 20 40 60 80 100
DTA DHL DMG DSO DPL DRF DVL DMH NTA NHL NMG NSO NPL NRF NVL NMH

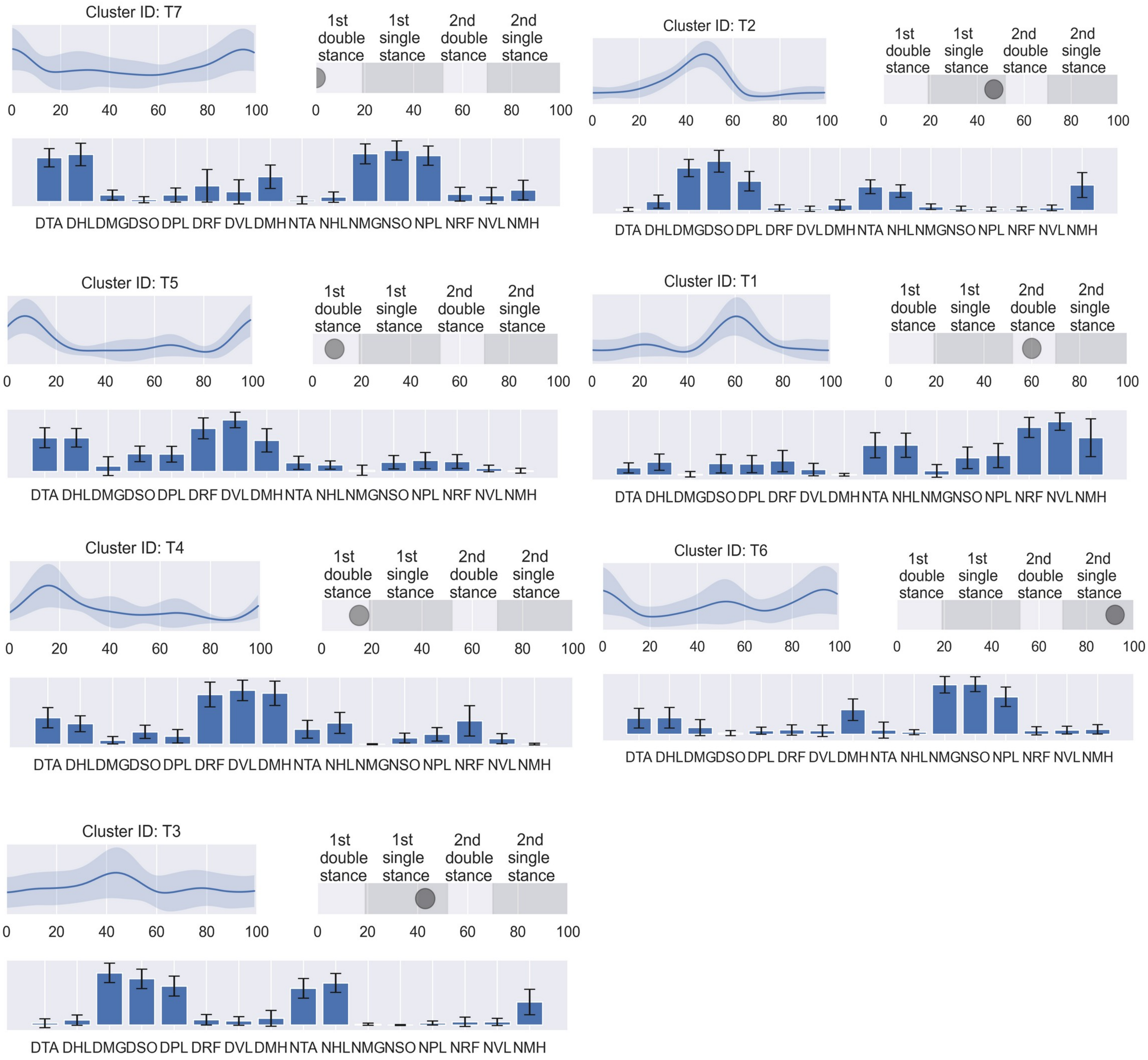
Cluster ID: T7
1st double stance
1st single stance
2nd double stance
2nd single stance
0 20 40 60 80 100
DTA DHL DMG DSO DPL DRF DVL DMH NTA NHL NMG NSO NPL NRF NVL NMH
Cluster ID: T2
1st double stance
1st single stance
2nd double stance
2nd single stance
0 20 40 60 80 100
DTA DHL DMG DSO DPL DRF DVL DMH NTA NHL NMG NSO NPL NRF NVL NMH
Cluster ID: T5
1st double stance
1st single stance
2nd double stance
2nd single stance
0 20 40 60 80 100
DTA DHL DMG DSO DPL DRF DVL DMH NTA NHL NMG NSO NPL NRF NVL NMH
Cluster ID: T1
1st double stance
1st single stance
2nd double stance
2nd single stance
0 20 40 60 80 100
DTA DHL DMG DSO DPL DRF DVL DMH NTA NHL NMG NSO NPL NRF NVL NMH
Cluster ID: T4
1st double stance
1st single stance
2nd double stance
2nd single stance
0 20 40 60 80 100
DTA DHL DMG DSO DPL DRF DVL DMH NTA NHL NMG NSO NPL NRF NVL NMH
Cluster ID: T6
1st double stance
1st single stance
2nd double stance
2nd single stance
0 20 40 60 80 100
DTA DHL DMG DSO DPL DRF DVL DMH NTA NHL NMG NSO NPL NRF NVL NMH
Cluster ID: T3
1st double stance
1st single stance
2nd double stance
2nd single stance
0 20 40 60 80 100
DTA DHL DMG DSO DPL DRF DVL DMH NTA NHL NMG NSO NPL NRF NVL NMH
